# The insect central complex as model for heterochronic brain development—background, concepts, and tools

**DOI:** 10.1007/s00427-016-0542-7

**Published:** 2016-04-07

**Authors:** Nikolaus Dieter Bernhard Koniszewski, Martin Kollmann, Mahdiyeh Bigham, Max Farnworth, Bicheng He, Marita Büscher, Wolf Hütteroth, Marlene Binzer, Joachim Schachtner, Gregor Bucher

**Affiliations:** Department of Evolutionary Developmental Genetics, Johann-Friedrich-Blumenbach Institute, GZMB, CNMPB, Georg-August-University Göttingen, Göttingen Campus, Göttingen, Germany; Institute of Medical Microbiology, Otto-von-Guericke-University, Magdeburg, Germany; Department of Biology, Animal Physiology, Philipps-University, Marburg, Germany; Department of Biology, Neurobiology, University of Konstanz, Constance, Germany

**Keywords:** Central complex, Brain, Heterochrony, Evolution, *Tribolium*, *Drosophila*

## Abstract

The adult insect brain is composed of neuropils present in most taxa. However, the relative size, shape, and developmental timing differ between species. This diversity of adult insect brain morphology has been extensively described while the genetic mechanisms of brain development are studied predominantly in *Drosophila melanogaster*. However, it has remained enigmatic what cellular and genetic mechanisms underlie the evolution of neuropil diversity or heterochronic development. In this perspective paper, we propose a novel approach to study these questions. We suggest using genome editing to mark homologous neural cells in the fly *D. melanogaster*, the beetle *Tribolium castaneum*, and the Mediterranean field cricket *Gryllus bimaculatus* to investigate developmental differences leading to brain diversification. One interesting aspect is the heterochrony observed in central complex development. Ancestrally, the central complex is formed during embryogenesis (as in *Gryllus*) but in *Drosophila*, it arises during late larval and metamorphic stages. In *Tribolium*, it forms partially during embryogenesis. Finally, we present tools for brain research in *Tribolium* including 3D reconstruction and immunohistochemistry data of first instar brains and the generation of transgenic brain imaging lines. Further, we characterize reporter lines labeling the mushroom bodies and reflecting the expression of the neuroblast marker gene *Tc*-*asense*, respectively.

**Electronic supplementary material:**

The online version of this article (doi:10.1007/s00427-016-0542-7) contains supplementary material, which is available to authorized users.

## Diversity of adult brain morphology and developmental timing

### The insect brain: morphological diversity based on a conserved architecture

**Fig. 1:**
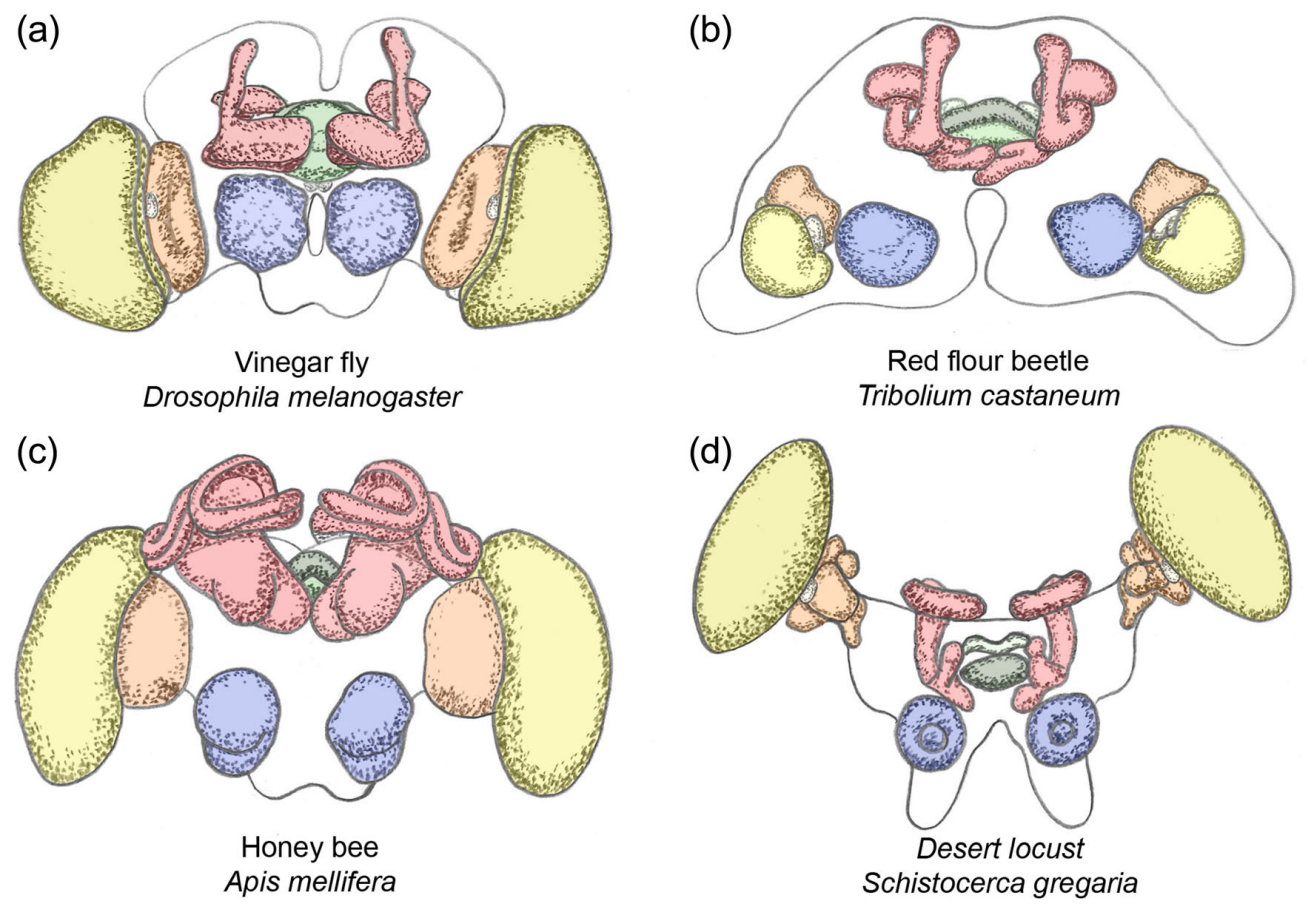
Diversity of adult insect brains. Shown are illustrations of the brains of the vinegar fly *Drosophila melanogaster* (**a**), the red flour beetle *Tribolium castaneum* (**b**), the bee *Apis mellifera* (**c**), and the desert locust *Schistocerca gregaria* (**d**). Based on (Rein et al. 2002; Kurylas et al. 2008; Dreyer et al. 2010; Rybak et al. 2010). All brains were sized to the same width and the respective neuropils have the same color code: *blue* antennal lobes, *red* mushroom bodies, *yellow* lamina of optic lobes, *orange* lobula of optic lobes, *green* central complex

The brain integrates sensory inputs, internal states, and other information to produce a specific behavioral pattern. Due to this essential role for survival, brain morphology and function are likely to be under high selective pressure. Indeed, the basic architecture of the insect brain is highly conserved and the different neuropils that serve particular functions are found in similar spatial arrangement in most adult insect species (Fig. 1) (Holmgren 1916; Hanström 1928; Snodgrass 1935; Weber 1966; Strausfeld 1976, 2005; Rein et al. 2002; Schachtner et al. 2005; Brandt et al. 2005; Kurylas et al. 2008; Homberg 2008; El Jundi et al. 2009a, b; Strausfeld et al. 2009; Dreyer et al. 2010). However, size and shape as well as timing of the development of the neuropils differ between insects. For example, 3D reconstructions of several insect brains revealed that the mushroom bodies of bees required for learning and memory have a large volume as compared to *Drosophila* and *Tribolium* (Brandt et al. 2005). Compared to the vinegar fly, the optic lobes (OLs) of the red flour beetle are small while its antennal lobes (ALs) are large (Dreyer et al. 2010). The combined relative volume of the central complex (CX) neuropils is larger in *Tribolium* as compared to *Drosophila* (for simplicity, we use the genus name of the model systems) (Rein et al. 2002; Brandt et al. 2005; Kurylas et al. 2008; El Jundi et al. 2009a; Dreyer et al. 2010). Interestingly, also, the timing of neuropil development varies. For instance, the CX is fully formed during embryogenesis in orthopteran insects while it develops postembryonically in flies. However, almost nothing is known about the genetic and cellular mechanisms that underlie the development of differences in morphology or developmental timing of neuropils between species. In this perspective paper, we present a novel developmental genetics approach to studying the development of homologous brain centers in different insect species. This approach has the power to reveal the cellular and genetic basis of insect brain diversification.

### The conserved developmental basis: neural lineages and genetic control

The genetic mechanisms underlying insect neural development have been studied most extensively in the vinegar fly *Drosophila melanogaster* and appear to be similar in other insects (Campos-Ortega and Hartenstein 1985; Skeath and Thor 2003; Urbach and Technau 2004; Brody and Odenwald 2005; Technau et al. 2006; Hartenstein et al. 2008; Egger et al. 2008). The developmental units that build the brain are neural lineages, which consist of one neuroblast (neural stem cell) and all its daughter cells, which can be either neurons or glia. The cell bodies of a given lineage stay together and the projection patterns of all daughter neurons are usually similar (Technau et al. 2006; Spindler and Hartenstein 2010; Pereanu et al. 2011; Boyan and Reichert 2011). In type I neuroblasts, asymmetric divisions produce several ganglion mother cells (GMCs), each of which divides once more to form postmitotic cells. Type II neuroblasts generate transit amplifying progenitors (TA-GMC; also called intermediate neural progenitors INP), which themselves divide in a stem cell mode giving rise to GMCs (Bello et al. 2008; Bowman et al. 2008; Boone and Doe 2008). Hence, many more daughter cells are produced per type II neuroblast. For instance, the *Drosophila* CX is formed from both types of neuroblasts (Bayraktar et al. 2010; Viktorin et al. 2011). Neural lineage development is not necessarily a continuous process. After an initial embryonic phase of division, many *Drosophila* neuroblasts enter quiescence for an extended period of time and resume proliferation at a later developmental stage (Egger et al. 2008).

Many aspects of cellular and genetic bases of neural development are conserved in animals (Denes et al. 2007; Hartenstein and Stollewerk 2015). In particular, the gene networks involved in specifying neural precursors and their spatial identity are highly conserved in insects and partly even in other arthropods (Wheeler et al. 2005; Stollewerk and Simpson 2005; Eriksson and Stollewerk 2010; Biffar and Stollewerk 2014; Stollewerk 2016). Homology of neural lineages has been suggested based on Crustacean neuroblasts, which show a comparable spatial arrangement, timing of delamination, transcription factor expression, and projection patterns as their insect counterparts (Ungerer and Scholtz 2008). Other examples are the similar location and the ongoing divisions of MB neuroblasts and the existence of type II neuroblasts at a comparable position and their contributions to the CX in a grasshopper (Boyan et al. 2010b; Boyan and Reichert 2011). In summary, at least the early development of the nervous system appears to be highly conserved.

## The question

The diversity of neuropil morphology and instances of heterochrony in brain development are well documented in a plethora of species. These differences between species must have evolved by modifications of the embryonic and postembryonic developmental programs. However, the nature of the changes of cellular and genetic mechanisms remains unknown. In part, this may be due to the fact that for technical reasons, developmental genetic studies have been largely restricted to *Drosophila* for a long time. We propose that the emerging tools for functional genetics outside *Drosophila* will allow comparing developmental mechanism between species.

## The concept: genome editing allows the genetic marking of homologous cells

In order to identify the differences in development between species, small groups of homologous cells need to be compared throughout development in both species, i.e., from the embryonic neuroblast to its progeny in the adult brain. There are several antibodies that allow marking for instance of serotonin-positive cells in different species. However, these markers do not mark neuroblasts or other neural progenitors during early phases of brain development. Furthermore, it has remained challenging even in *Drosophila* to mark a given lineage from the nascent neuroblast onwards and extensive enhancer trap collections marking subsets of neural cells are available only in *Drosophila*.

The recent development of genome editing tools changes the game because they have the potential to allow the genetic marking of groups of cells, which can be traced from neuroblast to the adult brain. By marking homologous cells in different taxa, the cellular basis of different developmental paths can be studied. We propose to establish such tools in a two-step process. First, groups of cells are genetically marked by their virtue of expressing a conserved transcription factor. Several transcription factors remain detectable in a certain lineage from the delaminating neuroblast to at least a subset of daughter cells of that neuroblast like engrailed (Kumar et al. 2009), and Ct, Dan, Dll, and Optix in type II neuroblasts (Bayraktar and Doe 2013). By genome editing, enhancer trap constructs encoding fluorescent protein can be integrated into the locus of such a neuroblast marker gene. The regulatory elements of that gene will drive expression of the fluorescent protein in all cells which express the gene. Transcription factors that are likely to be relevant should (1) be active in restricted regions in the neuroectoderm, (2) remain expressed in postmitotic neural cells of the brain, (3) be highly conserved in anterior brain development in bilaterians, and (4) their function should be required for the formation of the brain structure under study. Previous work identified good candidates: We and others showed that a set of conserved genes is required for patterning both vertebrate neural plate and invertebrate neuroectoderm (Lowe et al. 2003; Denes et al. 2007; Steinmetz et al. 2010; Posnien et al. 2011) and at least some of them were active in neural cells of the brain making them excellent candidates for marking homologous cells (Posnien et al. 2011).

In a second step, homology of marked cells needs to be corroborated by comparing location of marked cell bodies, projection patterns, neuromodulator content, and other features. A similar reasoning provided the basis of previous efforts to identify homologous neural cells based on molecular similarity (Urbach and Technau 2003; Arendt 2005; Tomer et al. 2010; Biffar and Stollewerk 2014). In addition, the assumed continuous expression from neuroblasts to the postmitotic cells will have to be shown for each reporter. The methods outlined here should be applicable to study the stage and the nature of developmental changes that lead to different morphologies or to differences in developmental timing. In the following, we describe an intriguing case of heterochronic development, which could be studied using this approach.

## The central complex as a model for studying brain evolution

### The central complex—a higher order integration center of the brain

**Fig. 2:**
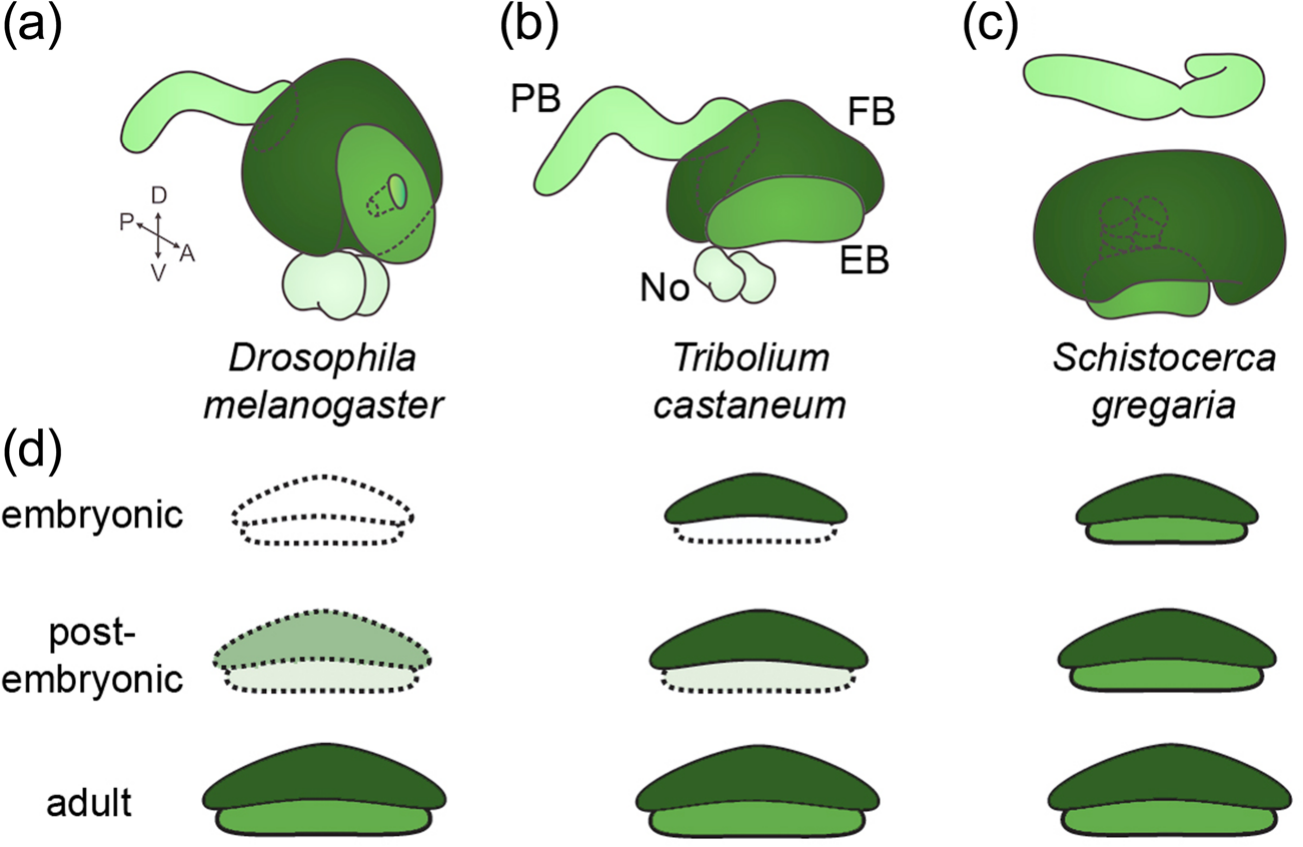
Heterochronic development of the central complex. **a**–**d** The central complex of adult specimen of the vinegar fly *Drosophila melanogaster* (**a**), the red flour beetle *Tribolium castaneum* (**b**), and the desert locust *Schistocerca gregaria* (**c**) are shown sized to the same width. Note that the overall architecture of the CX components is similar as is their basic connectivity (not depicted). Central body with fan-shaped body (*FB*) and ellipsoid body (*EB*). *No* noduli; *PB* protocerebral bridge. **d** Heterochronic development of the CB is depicted schematically for the species (**a**–**c**). This neuropil is fully developed in desert locust hatchlings, representing the ancestral condition. In the beetle, only the FB (*dark green*) is present while the EB (*light green*) is added postembryonically. In the vinegar fly, the neuropil becomes apparent only at late larval and metamorphic stages. *Light colors* and *hatched outlines* mark neuropils that are developing but not yet functional while *white* indicates lack of detectable neuropil structure. Note that neuropil shapes are unified and other CX neuropils have been omitted for simplicity. **a**–**d** Redrawn from (Hanesch et al. 1989; Dreyer et al. 2010; Kaiser 2014)

We propose that the insect central complex (CX) is an excellent case to study heterochrony as one aspect of evolutionary adaptation of the brain. Before doing so, we briefly introduce to morphology and function of this neuropil—please refer to the recent review by Pfeiffer and Homberg for original work (2014). Apart from the paired noduli, the insect central complex (CX) typically consists of a set of unpaired neuropils spanning the midline (i.e., the protocerebral bridge (PB), central body (CB) with upper and lower unit, also called fan-shaped body (FB) and ellipsoid body (EB)) (Fig. 2a–c). The paired lateral accessory lobes (LAL) are not part of the CX but they are considered as strongly associated to the CX (Homberg 2008). The wiring scheme within and between these neuropils is highly ordered and reflects their organization in columns and layers. For instance, tangential neurons connect the columns within one neuropil while columnar neurons connect specific columns of different neuropils with each other while other neurons connect the CX to other brain parts (Power 1943; Williams 1975; Strausfeld 1976; Homberg 1985, 2008; Hanesch et al. 1989; Loesel et al. 2002; Boyan and Williams 2011; Pfeiffer and Homberg 2014). Among other higher order brain functions, the CX is involved in sky compass orientation, locomotor behavior, courtship, and memory among others (Strauss 2002; Homberg 2008; Pfeiffer and Homberg 2014; Heinze 2015). Similar midline neuropils have been found in many Arthropoda including Collembolans, Crustacea, Chelicerata, Myriapoda, and even Onychophora (Loesel et al. 2002; Strausfeld et al. 2006; Kollmann et al. 2011), and homology of the CX with the vertebrate basal ganglia has been suggested (Strausfeld and Hirth 2013). The function of the CX has become a major focus of neurobiology research and extensive imaging and misexpression resources are being generated (Hanesch et al. 1989; Loesel et al. 2002; Pfeiffer et al. 2008; Cardona et al. 2010; Young and Armstrong 2010a, b; Jenett et al. 2012; Takemura et al. 2013; Ito et al. 2014). Several neural lineages contributing to the CX were found but the embryonic part of their development remains poorly studied (Renn et al. 1999; Young and Armstrong 2010a, b; Pereanu et al. 2011; Riebli et al. 2013; Yang et al. 2013).

### Heterochronic development of the central body

An intriguing divergence was observed regarding the timing of CX development, which was comprehensively described for the CB (Hanström 1925; Panov 1959; Wegerhoff and Breidbach 1992; Boyan and Williams 1997, 2011; Loesel et al. 2002; Boyan and Reichert 2011). In most hemimetabolous insects, the CB develops fully during embryogenesis. During postembryonic development, the CB just grows in size with minor morphological changes. This has been shown for several orthopteran taxa including crickets (*Gryllus*, *Metrioptera*), grasshoppers (*Calliptamus*, *Schistocerca*), mantids (*Ameles*), and stick insects (*Dixippus*—now *Carausius*). Likewise, this has been found in hemipteran bugs (*Palomena*). By contrast, a CB neuropil is morphologically not detectable in hatchlings of some butterflies and moths (*Pieris*, *Ephestia*), mayflies (*Ephemera*), hymenoptera (*Apis*), and flies (*Musca*, *Drosophila*) (Panov 1959). However, in *Drosophila* first instar larva, some commissural tracts prefigure the location of the later CB (Hinke 1961; Riebli et al. 2013). A partial CB, namely the FB, was found in hatchlings of some Neuroptera (*Chrysopa*, *Ascalaphus*), Lepidoptera (*Antheraea*, *Manduca*), and a Diptera (*Culex*) (Panov 1959; Homberg and Hildebrand 1994). Within beetles, there are species where hatchlings do not yet display a CB like the curculionid *Anthonomus* or *Oryctes* (Jawlowski 1936) while a partial CB was detected in tenebrionid beetles (*Tenebrio molitor*, *Tribolium castaneum*) (Wegerhoff and Breidbach 1992; Wegerhoff et al. 1996; this work). The partial CB present in hatchlings usually represents the fan-shaped body. In summary, the timing of CB development has repeatedly been shifted relative to overall development of the animal during insect evolution with the full embryonic development probably being ancestral. Such evolutionary shifts of developmental timing have been termed “heterochrony” (Gould 1977). The different times of CB emergence within insect families like flies and beetles indicates that heterochrony has evolved several times. The functional relevance remains speculative: Most species where hatchlings lack a CB show no or only a poorly developed visual system (Panov 1959) and the embryonic development of the CX coincides with the emergence of walking legs—the legs are fully developed in hatchlings of hemimetabolous insects while tenebrionid larvae have reduced legs and *Drosophila* larvae do not have any legs (Pfeiffer and Homberg 2014).

## Cricket, beetle, and fly as model systems to study the genetic basis of brain evolution

What insect model systems are likely to be most useful for research on the functional genetics of brain evolution? Of course, these model systems need to represent clear evolutionary changes of neuropil size, shape, or timing of development but they need to be amenable to functional genetics as well. Given these demands, the cricket *Gryllus bimaculatus*, the vinegar fly *D. melanogaster*, and the red flour beetle *T. castaneum* form an excellent group of model organisms. Functional genetic tools within orthopterans are best developed in the cricket *Gryllus* with RNAi and transgenesis established and it represents the ancestral state of CX development (Fig. 2d) (Panov 1959; Miyawaki et al. 2004; Nakamura et al. 2010; Zeng et al. 2013; Watanabe et al. 2014). Cellular development of the ancestral state of the CX has been described best in the orthopteran *Schistocerca gregaria* (Boyan et al. 2003, Boyan et al. 2010a, b; Williams et al. 2005; Boyan and Reichert 2011; Boyan and Williams 2011; Boyan and Liu 2014). However, due to their close phylogenetic relationship, it is likely that most knowledge gained in *S. gregaria* will be transferrable to *G. bimaculatus*.

Tenebrionid beetles represent the intermediate state where the FB forms during embryogenesis. It is only during the pupal stage that the ellipsoid body is eventually completed (Wegerhoff and Breidbach 1992; Wegerhoff et al. 1996) (Fig. 2d). Importantly, with respect to functional genetics, the red flour beetle *Tribolium* is second only to *Drosophila* including transgenesis, large-scale enhancer trap screen, misexpression tools, and in vivo imaging lines (Berghammer et al. 1999; Lorenzen et al. 2003; Trauner et al. 2009; Schinko et al. 2010; Posnien et al. 2011; Schinko et al. 2012; Sarrazin et al. 2012). RNAi-mediated gene knockdown is strong and is either environmental or systemic. Hence, when dsRNA is injected into the hemolymph, the knockdown spreads to reach all cells of the injected animal and is even transmitted to the offspring of injected females (Brown et al. 1999; Curtis et al. 2001; Bucher et al. 2002; Tomoyasu and Denell 2004; Miller et al. 2012; Peel et al. 2013). Mutant phenotypes were described for *Tc*-*knirps*, *Tc*-*Distal*-*less*, and *Tc*-*sex combs reduced* and in all these cases, RNAi phenocopied the null phenotype. Resources for large-scale RNAi screening are being established with currently half of the genome being covered by dsRNA templates (Dönitz et al. 2015; Schmitt-Engel et al. 2015). Finally, the CRISPR/Cas9 system has been established (Gilles et al. 2015).

*Drosophila* represents the most derived state of CX morphology, with the CX anlagen of the hatchling consisting of commissural tracts lacking neuropil morphology and synapses indicating non-functionality (Fig. 2d). Only during late larval stages and metamorphosis, the CX neuropils develop and mature (Renn et al. 1999; Young and Armstrong 2010a; Pereanu et al. 2011; Riebli et al. 2013; Pfeiffer and Homberg 2014). *Drosophila* is the prime model system for insect functional genetics with an excellent toolkit. Due to the delayed development in the fly, research on CX formation is focusing on the postembryonic phase (Viktorin et al. 2011; Jiang and Reichert 2012; Carney et al. 2012; Bayraktar and Doe 2013; Yang et al. 2013).

## Studying brain development in the red flour beetle

### Immunohistochemistry confirms presence of one central body neuropil in the L1 larval brain

**Fig. 3:**
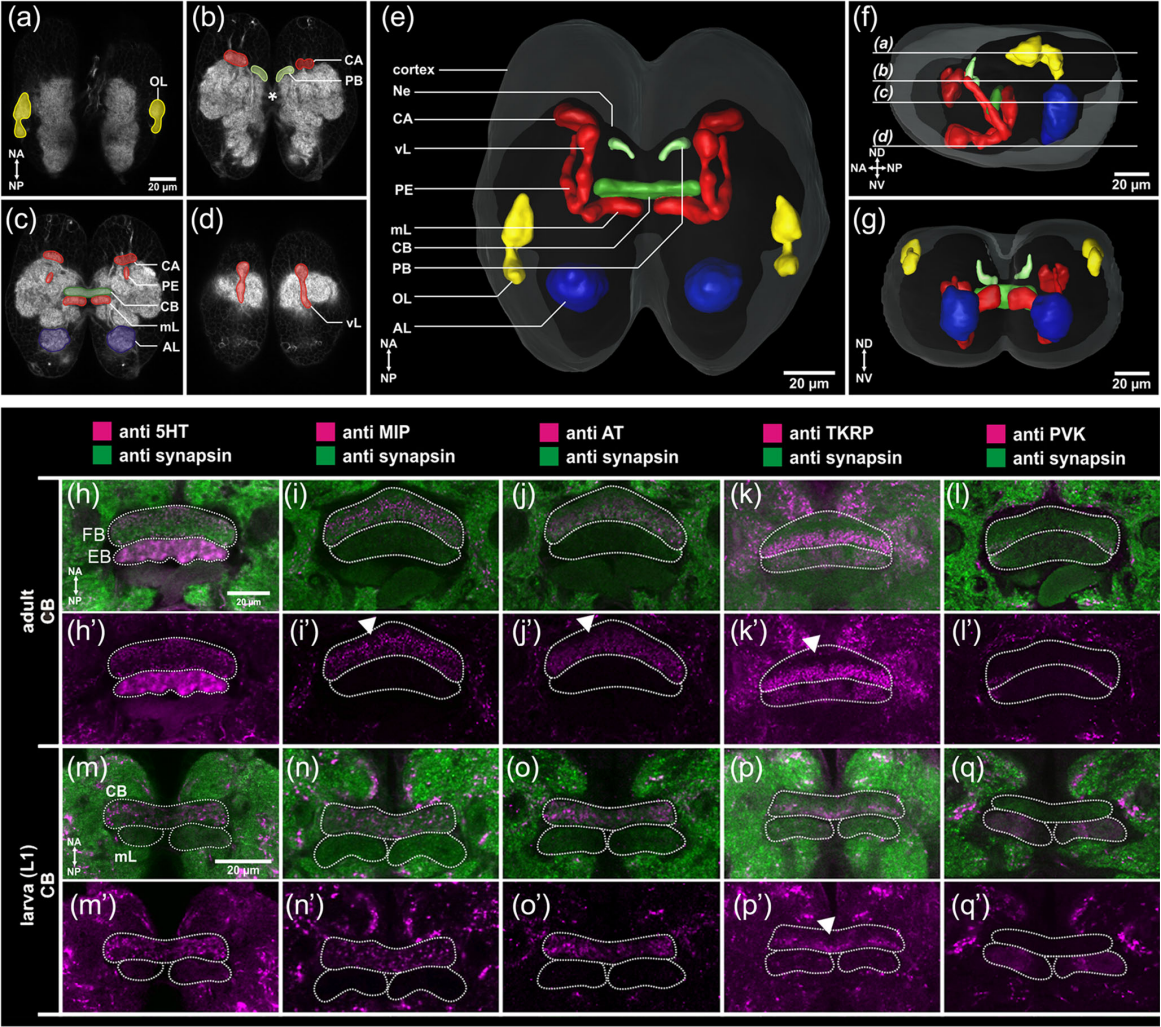
The first instar larval brain of *Tribolium castaneum* and expression of neuromodulators in the CB. **a**–**d** Dorsal view of a first instar larval brain stained with an antibody detecting synapsin. The level of the sections is displayed in the reconstruction (**f**). **e**–**g** 3D reconstruction of neuropils based on the synapsin staining (**a**–**d**). Color code: *blue* antennal lobes (*AL*), *yellow* (anlagen of the) optic lobes (*OL*), and *red* mushroom bodies (*MB*) with calyx (*CA*); *PE* pedunculus, *vL* vertical lobe, and *mL* medial lobe. The cortex layer containing most cell bodies is shown in *light gray*, while the entire neuropilar mass of the brain is shown in *dark gray* (*Ne*). **h**–**q** Optical sections through the CB in adults (**h**, **l**) and first instar larvae (**m**, **q**) stained against serotonin (*5HT*), myoinhibitory protein (*MIP*), allatotropin (*AT*), tachykinin-related peptide (*TKRP*), and periviscerokinin (*PVK*). Neural anterior (n-anterior; *NA*) is up in all panels. *FB* fan-shaped body, *EB* ellipsoid body. The staining in the larval CB resembles staining of the FB in adult brains, corroborating the previous assumption that only the FB develops during embryogenesis. The anterior rim of the adult FB lacks MIP and AT expression (*white arrowheads* in *i*ʹ and *j*ʹ), which is not the case in larval CB. An n-anterior lack of TKRP reactivity, in contrast, is found in both larval and adult brains (white arrowhead in *k*ʹ and *p*ʹ). *Scale*- and *orientation bars* (**a**) account for (**b**–**d**), *bars* (**h**, **m**) account for *i*–*l*, *n*–*q*, *h*ʹ–*l*ʹ, and *m*ʹ–*q*ʹ

*Tribolium* is a useful model for CX development for two reasons: first, it allows studying the embryonic aspects of CX development. Second, comparison to *Drosophila* will reveal the cellular and genetic basis of the heterochronic shift between these species. In order to establish *Tribolium* as a model system for CX development, we first studied the L1 brain morphology by immunohistochemistry targeting synapsin followed by 3D reconstruction (Fig. 3; see Online Resource S1 for methods). We found the upper division of the central body (FB; dark green in Fig. 3a–g) with a flattened bar-like shape. An EB was not found at that stage. These data are in line with findings in *T. molitor* where the larval CB was described to consist of the FB only. The PB (Fig. 3b, e–g; light green) was present but medially split, probably with some fibers connecting both parts (Fig. 3b; asterisk). Only during later larval development these two parts fuse (not shown). Interestingly, a PB was not detected in hatchlings of *T. molitor* but a split PB emerged at later larval stages before it fuses during metamorphosis (Wegerhoff and Breidbach 1992; Wegerhoff et al. 1996). Whether this reflects a heterochronic shift within Tenebrionidae or is due to different sensitivity of the methods used remains to be tested. We did detect no noduli in L1 larval brains.

Using immunohistochemistry, we found the biogenic amine serotonin (5HT) as well as the neuropeptides myoinhibitory peptide (MIP), allatotropin (AT), and tachykinin-related peptide (TKRP) in both the adult FB and the L1 CB. The neuropeptide periviscerokinin (PVK) was absent in the adult and larval CBs (compare Fig. 3h–k with hʹ–kʹ; see Online Resource 1 for more detailed description and Fig. S1 in Online Resource 2 for comparison of other neuropils and Online Resources 3–14 for confocal stacks of adult and embryonic brains) (Wegerhoff and Breidbach 1992). Curiously, the n-anterior rim of the adult FB lacks MIP and AT immunoreactivity (white arrowheads in Fig. 3iʹ and jʹ) while in the larval CB, a correspondingly unstained rim was not found. In case of TKRP, n-anterior immunoreactivity lacked in both adult and larval CB (white arrowheads in Fig. 3kʹ and pʹ). The expression of synapsin and neuromodulators in the CB strongly indicates that the neuropil is functional in the hatchling already. As proof of principle, we tested several stainings in *Tc*-*six3* RNAi-knockdown animals, where CB deletion had previously been described (Posnien et al. 2011). The brains of L1 larvae were dissected and the previously published MB phenotype was confirmed by immunohistochemistry using the DC0 antibody (see Fig. S3 in Online Resource 2). Immunohistochemistry in *Tc*-*six3* RNAi-knockdown animals against DC0, 5HT, and MIP showed a specific signal in the brain as in wild type but the signal corresponding to the CB was not found confirming our previous data (see Fig. S3 in Online Resource 2).

To increase the repertoire of neural markers in *Tribolium*, we tested a number antibodies used in *Drosophila* research but most of them showed no or inconsistent signal (see Online Resource 16 for complete list). However, only the antibodies targeting reversed polarity (a marker for glia), aPKC, Bazooka (both asymmetrically loalized in neuroblasts), even skipped, engrailed (markers for subsets of neuroblasts and neurons), fasciclin 2 (marker for subsets of axons), death caspase 1 (marker for apoptosis), and phospho-histone-3 (marker for dividing cells) were confirmed to cross-react.

### Transgenic lines marking the mushroom bodies and reporting *asense* expression

**Fig. 4:**
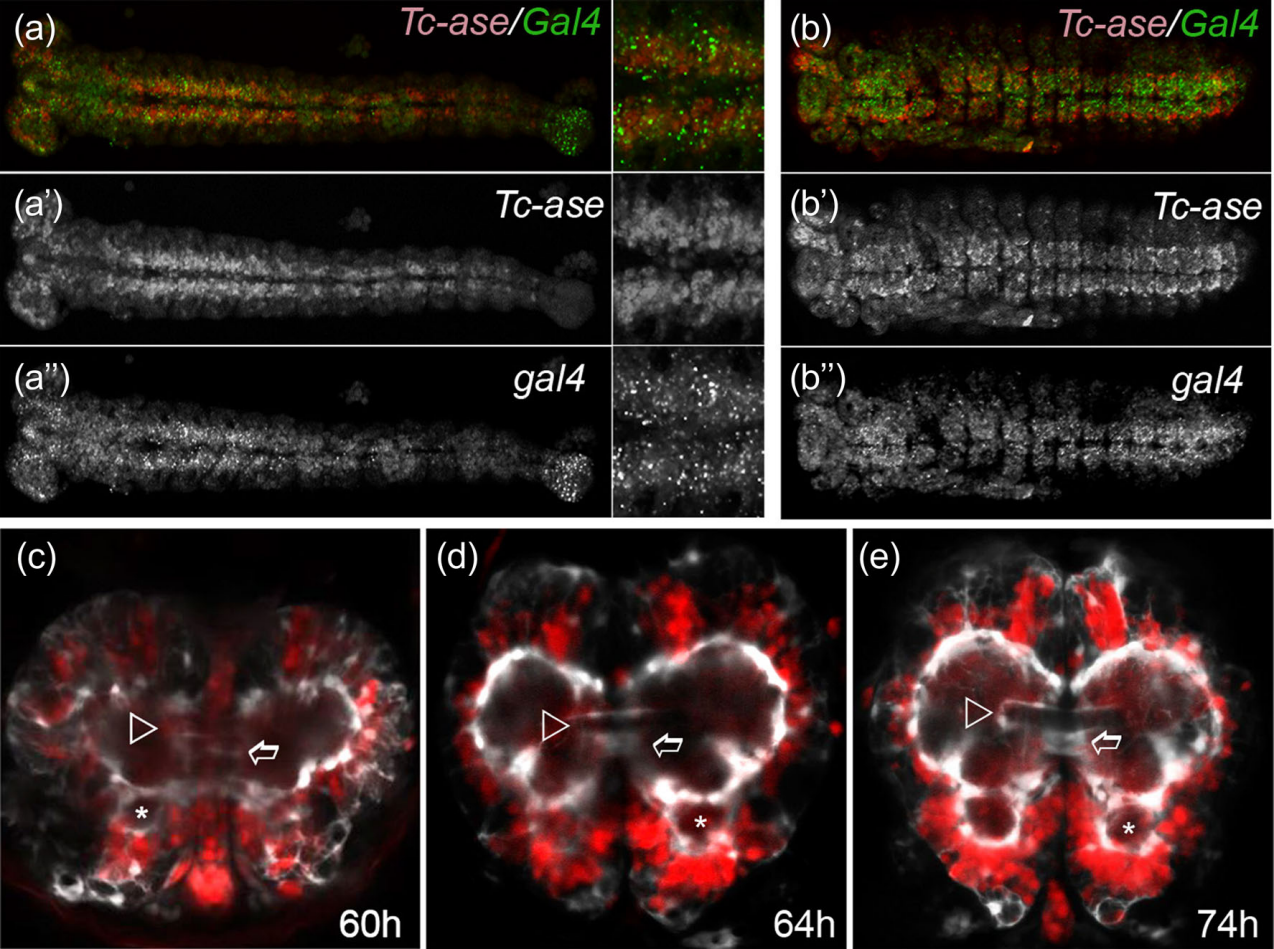
Transgenic in vivo imaging reporters for brain research in *Tribolium castaneum*. **a**, **b** The regulatory region of *Tc*-*asense* drives expression of Gal4 in neuroblasts. The overlap of expression was confirmed with double in situ hybridization detecting *Tc*-*ase* (*red*) and *gal4* (*green*). **a**–**a**″ mid-embryogenesis; **b**–**b**″ late embryogenesis. **c**–**e** Developmental series of the fluorescence signal of the brainy line. The ECFP signal marking glia is shown in *white* while the DsRed-Express signal in neurons is depicted in *red*. Embryos are oriented n-anterior to the top. *White arrowhead* marks the developing central body; *stars* indicate the antennal lobes while *open arrows* mark the median lobes of the mushroom bodies. **d**, **e** The glial sheath of the central body is first detected in 60–64-h-old embryos and is clearly visible in brains of hatchlings (**e**). See Online Fig. S2 in Resource 2 for earlier stages

For the analysis of cell body location and projection patterns in wild-type and knockdown phenotypes, it is advantageous to have transgenic lines that mark specific subsets of neural cells or certain neuropils. From a previous enhancer trap screen (Trauner et al. 2009), we identified the line G11410 which marked the mushroom bodies with enhanced green fluorescent protein (EGFP) (“MB-green”) (Fig. S2 in Online Resource 2) (Posnien et al. 2011; Binzer et al. 2014). Colocalization of EGFP with DC0 in the neuropil confirmed that MB was marked (Skoulakis et al. 1993; Farris and Strausfeld 2003) (Fig. S2 in Online Resource 2). In order to mark neuroblasts (NBs), we used an intronic fragment of the NB marker *Tc*-*asense* (see Online Resource 1 for sequence) to drive Gal4delta (“ase-Gal4”) (Wheeler et al. 2003). Indeed, when crossed with the UAS-tGFP line, fluorescence was detected in the ventral nerve cord and the brain of late embryos (not shown). Double in situ hybridization confirmed co-expression of *Gal4delta* and *Tc*-*asense* (Fig. 4a, b).

### Reporter lines for neural and glial cells allow in vivo imaging of brain development

We wanted to generate imaging lines marking neural cell types. An artificial promoter containing three eyless/Pax6 binding sites (3XP3) and the *Drosophila* heat-shock core promoter drove EGFP in the eyes in a wide range of animals (Sheng et al. 1997; Berghammer et al. 1999). However, recent work indicated that 3XP3 reports the expression of the homeobox gene *Pph13* rather than the *eyeless*/*Pax6* (Mishra et al. 2010). In *Tribolium*, we noted additional fluorescent signal in the brain with variable intensity. To render this signal more robust, we generated transgenic lines where six copies of the P3 elements (6XP3) drove either enhanced cyan fluorescent protein (ECFP) or dsRed-Express from the *Tribolium hsp68* core promoter. Both constructs led to a specific signal in glial cells (Posnien et al. 2011). The respective lines were called “glia-blue” and “glia-red.” To determine the portion of marked glia, we tested for colocalization with the glia marker reverse polarity (4α3, Repo) (Fig. S2 in Online Resource 2) (Xiong et al. 1994). We found a large degree of co-expression in the nuclei of glia cells while the reporter additionally visualized the cell bodies. Quantification (excluding the OL) revealed that about 608 glial cells were marked by the Repo antibody (*n* = 5; SD = 64; SE = 32) with about 524 (86 %) of them being positive for the ECFP signal as well (*n* = 5; SD = 47.5; SE = 23.8). About eight ECFP-positive cells (1.5 %) did not show Repo staining (*n* = 5; SD = 2.9; SE = 14). These could be glia of mesectodermal origin, which are Repo negative, at least in *Drosophila* (Parker and Auld 2006).

“Neuron-red” is a transgenic line where the upstream region of one of the *Tc*-*EF1*-*alphaB* paralogs unexpectedly drove DsRed-Express in neurons (Averof, personal communication; see Online Resource 1 for sequence) (Posnien et al. 2011). To monitor both glia and neurons in the same animal, we crossed the glia-blue and neuron-red lines establishing the “brainy” line (Fig. 4 and Fig. S2 in Online Resource 2; glia in white, neurons in red) (Posnien et al. 2011). To test the suitability for in vivo imaging, we monitored both signals in living embryos. Expression of ECFP was first detected in 17-h-old embryos (32 °C) while first expression of DsRedEx became detectable at 24 h (Fig. S2 in Online Resource 2). Strong branching of glia was observed in 40-h-old embryos. First signs of the CB were detected from 55 h onwards (open arrowhead in Fig. 4c–e). Antennal lobes (AL) and the median lobes of the mushroom bodies (mL) were detected from 60 h onwards (asterisks and open arrow in Fig. 4c–e, respectively).

## Conclusion

The cellular and genetic mechanisms of the evolution of the tremendous diversity of neuropil morphology remain enigmatic. Using the models, concepts, and tools presented in this work, it will be possible to study this fascinating question. Further, we believe that the central complex is an excellent model system to study heterochrony as one aspect of brain evolution and we present tools that will turn out to be useful for the study of this and additional questions regarding the brain development of *Tribolium*.

## Electronic supplementary material

Below is the link to the electronic supplementary material.

ESM 1 Colocalization of repo with 6XP3-signal, colocalization of DC0 in the MB line and time course of CB development. (PDF 717 kb)

ESM. 2 Analysis of *Tc*-*six3* phenotype by immunohistochemistry (PDF 1117 kb)

ESM. 3 Synapsin larva stack (AVI 4469 kb)

ESM. 4 DC0 larva stack (AVI 847 kb)

ESM. 5 5HT larva stack (AVI 824 kb)

ESM. 6 5HT adult stack (AVI 1265 kb)

ESM. 7 MIP larva stack (AVI 677 kb)

ESM. 8 MIP adult stack (AVI 1281 kb)

ESM. 9 AT larva stack (AVI 1082 kb)

ESM. 10 AT adult stack (AVI 888 kb)

ESM. 11 TKRP larva stack (AVI 797 kb)

ESM. 12 TKRP adult stack (AVI 994 kb)

ESM. 13 PVK larva stack (AVI 706 kb)

ESM. 14 PVK adult stack (AVI 957 kb)

ESM. 15 glia-blue line ECFP and Repo (AVI 571 kb)

ESM. 16 Table of all antibodies tested for cross-reactivity in *Tribolium*. (*XLSX 13 kb*)
