# Supplementary material for: The insect central complex as model for heterochronic brain development—background, concepts, and tools
Source: Dev Genes Evol. 2016 Apr 7;226:209–19. doi: 10.1007/s00427-016-0542-7 (PMC4896989; doi:10.1007/s00427-016-0542-7)
Supplement: Supplementary file 1 — Colocalization of repo with 6XP3-signal, colocalization of DC0 in the MB line and time course of CB development. (PDF 717 kb) [file 427_2016_542_MOESM1_ESM.pdf]

**The insect central complex as model for heterochronic brain development – background, concepts and tools**

Nikolaus Dieter Bernhard Koniszewski[1/2]*, Martin Kollmann[3]*, Mahdiyeh Bigham[1], Max Farnworth[1], Bicheng He[1], Marita Büscher[1], Wolf Hütteroth[3/4], Marlene Binzer[3], Joachim Schachtner[3], Gregor Bucher[1#]

[#]Corresponding author: Gregor Bucher

# Details on L1 brain morphology

## Anatomy of the brain of the first instar larva

Four pronounced neuropils could be identified in the first instar larva: We characterized the unpaired larval central complex (CX) and the paired mushroom bodies (MBs), antennal lobes (ALs), and optic lobes (OLs) (Fig. 3 a-g). The L1 CX consists of a central body (CB) with the upper unit (CBU), and the protocerebral bridge (PB), which is split into two parts connected by fibers. The lower unit of the CB (CBL) and the paired noduli (NOs) are missing. The MBs consist of the calyces (CAs) and the pedunculi (PEs) consisting of the median and vertical lobes (mL and vL), similar to adult animals. The larval ALs contain about 40 - 50 glomeruli, while the adults ALs have about 70-90 glomeruli per AL (Dreyer et al. 2010). The OLs exist only as anlagen and are differentiated in a distal and a proximal part.

## Neuromediators in neuropils of larval and adult brains

Antibodies against neuromediators are known to stain distinct areas of adult insect brains. Here we discuss the similarities and differences of observed stainings in larval (L1) and adult brains of *T. castaneum* based on Fig. 3 h/h'-q/q' and supplementary Fig. 1 (this work) and previously published stainings of the adult ALs and MBs [1,2].

<u>Central body</u>

The antibodies against 5HT, MIP, and AT produced homogeneous scattered staining in both the adult and larval CBU (Fig. 3 h-j, h'-j' and m-o, m'-o'). Different from the CB in L1 larvae, the adult CBL is intensely labeled by the 5HT antibody. This supports the hypothesis that the CBL is absent in first instar larva, as described for *Tenebrio molitor* [3,4]. The antibody against TKRP predominantly stained the n-posterior part of the CBU in both adults and larvae (Fig. 3 k, p and k', p'). Hence, the staining patterns appear to be maintained from larval to the adult form. We also observed some differences: The distinction between MIP and AT positive and negative labeled regions in the adult CBU (Fig. 3 i' and j'; arrowhead) was not reflected in the larval pattern. Further, the staining with the PVK antibody revealed staining in a small part of the n-posterior-lateral CBU, while the larval CBU is devoid of any PVK staining.

<u>Mushroom bodies</u>

Adult and larval MBs show a high degree of overall similarity in the neuromediator staining patterns but differences are found as well. The 5HT antibody labels a scattered network of extrinsic fibers in the CAs of both stages. However, the other parts of the MBs do not show any staining in larvae while in the adult diffuse and homogeneous staining is found (Fig. 3 m, m’, supplementary Fig. S1 p, p' and u-

x). The MBs were devoid of MIP and AT stainings in both adults and larvae except for a very small area near the junction of the PE, vL and mL in adult animals [1] (supplementary Fig. S1 q, q', r, r').

In adult animals the TKRP antibody labels mainly the inner core of the MB but also scattered areas of the PE, mL, and vL [1]. In the larva, the scattered patterns are found as well but a TKRP immunoreactive core was not found (supplementary Fig. S1 s, s'). This indicates that the neurons homologous to ab core neurons in Drosophila differentiate later during development [5,6].

The antibody against PVK showed no immunostaining in the MBs of adults [1]. By contrast, the larva showed immunoreactivity in a restricted area, resembling a belt around the middle of the mL, as well as at the distal tip of the vL (Fig. 3 q, q' and supplementary Fig. S1 t, t').

Optic lobes

The anlagen of the larval OLs of *T. castaneum* can be differentiated into a distal and a proximal part (Fig. 3e), while the adult OLs are composed of the lamina (LA), medulla (ME), lobula (LO), lobula plate (LOP), and accessory medulla (AME). Because the LA is often damaged during preparation, data of the LA are not included in this work. The differences of the larval and adult OLs make a comparison difficult. In the larva, staining with the 5HT and MIP antibody labeled the entire proximal part of the OL anlagen (supplementary Fig. S1 f, f', g, g'). Adult OLs showed immunoreactive areas in all parts (supplementary Fig. S1 a, a', b, b'). The antibody against AT did not label the OLs in neither larva nor adult (supplementary Fig. S1 c, c', h, h'). The antibodies against TKRP and PVK clearly stained distinct areas of the proximal part of the larval OL anlagen (supplementary Fig. S1 i, i', j, j'). In the adults however, the antibody against TKRP labeled the ME, LO, and LOP as well as faintly the AME (supplementary Fig. S1 d, d'), while the antibody against PVK labeled only the AME faintly (supplementary Fig. S1 e, e').

Antennal lobes

The overall anatomy of the larval and adult ALs are similar. Both are ball-shaped and composed of spherical glomeruli. However, in L1 there are about 40-50 glomeruli while in adults there are about 70 glomeruli (Dreyer et al. 2010). Immunostainings with the antibodies against 5HT and MIP stain the entire AL in the larva and in the adult (supplementary Fig. S1 k, k', l, l', y, and [1]). It seems that in both stages, the 5HT antibody labeled branches throughout the ALs, innervating only the surface of the glomeruli, while the MIP antibody labeled the entire volume of the glomeruli. The AT antibody produced a scattered staining across all glomeruli of the ALs in both stages (supplementary Fig. S1 m, m'; [1]). The labelling by the antibody against TKRP differed drastically between larva and adult. In adults, all glomeruli of the ALs were clearly labeled, while in the larva only a faint, scattered staining of the ALs was observed (supplementary Fig. 1 n, n' and [1]). The antibody against PVK labeled scattered spots between the glomeruli in the posterior portion of adult AL [1]. In contrast, the larval ALs seem to be devoid of any PVK immunoreactivity (supplementary Fig. 1 o, o').

## Tc-asense regulatory region

The following regulatory sequence of the 1$^{st}$ intron of the *Tc-asense* gene drives expression in the pattern described in Fig. 4a,b.

**Primer with overhang and restriction site (5´-3´)**

GTGACTGGATCCACAAATAAACCGCTTTTGAAATGC

GTCAGTGGTACCGTCACTACTGTCTGTGTCAG

ACAAATAAACCGCTTTTGAAATGCTTTCCTGCTCGTTCCCGCACCTAGACCAGGAGCCAATTGAAAATAAACAA
TCAGAAGATAAAAAGACTAGAAATCGCTCAAAAGATTGCAAAACCCCTCTAAAAATTATCCCCAAATTCAATTA
CAACTACGTAGTTTTAGAAAAAATACAGGAGTTTCCTTGAAATTTTAGTGGTGATGCGGTATGTTTTCTCAGTCT
CTGATATAATTAGTTTTTAAAAAAAGGTCAATGCTAGTTTTTGTAATGTAAGGAAGAGATAATCTTAGACACTT
GTAGCAACATCCCTCTCTCTAACAATGATATTTATCCTAATGTTCCAATTTTTCTTGTTGTCTTAAATTTTTAAATA
GTCGCTGAGGAAGTGCTGATGTTGAAGCCCGAAACACAAGCATTCCAAAACATTTAAACCAGTAAGCCTTGAT
TGGTTATACTTTTCAAAAATGTCTCTACAAGAATCTGGTTTCCAATTGGATATTAATCTGATGGCTCTTTTTTATT
AAAATTAAGAAAAACACAATTAAATTAACTTTATTAAAATTAAGAAAAATCTCTTCAAAAAATAATACGTACCAT
ATTCTCTCACACATTGGAAGATAAGCGTAATCTATTTTTTTATTCTTTTCACACATTCAGTGTTGAGTTTTCGTGT
TATAACTCTTTCTATATTAGATATTCATGTAAAGTAATTCCTCGAGATATTAGTATTCGATAAAACAGTAAAATT
ATTATTATTCTAGGGTTGATTTCAATCGATGGAATGTATTAGAAATTCGCTTTCTCAGAGAGACTTTTTTGAAGA
AAATGCAACTGTTAGAAAAATTTTAATTACAGAACACTTTGCTAGATTATATAAAAACTAACATTTGTTCTTTAA
TTTTTGTTCCCAATTCCAAAAGCTTCATTTTTTTGGTATTCTCAATTACCGCAATTATTTTTCTGTAATAAGAAGTC
ACTGCTCTTATTTTTAATTTTTAAACTAATTTAGGAATGTCGTCATAAACTAATTCTTCTATTATGTCTGGTTTTAT
ACCTCAAATTGGAGCATATATTAAGATGTTAAGTAAATTCCCACCCAATTTAAAATTTTTATTTAGATCAAAATG
CCTATCGGCCTGACGGCCTTCACCAGTTCTTCGTCAAAACACTTCACATATAATTATTGTAATTAAAATCAAACA
TTATTACATTAAACACTTAAAATCAAGATTAAACTTTAAAATTTGTTTGCCCTTTCTTTTCTTTGACAGCCATTTGT
GCCGAAACCGGTATTCTGATCTAAATAAAAATTTAAAATTGGGAGGAATTTTACTTCAGACATGTACTTATAGC
GATTAAAACTACTTGTCTACTACTCAAGTATAGTGATTAAAACTAGTATTTTCTTTTACCAGAATTTTTTATTCTC
CAAAAGTAATTCAACATTATAATAAAATAACTACTTAGATTATCAGCTTACATCCCTATTGCATTTAAAAGAGTT
TTCTAAATCCATGACTTCTCGTAATTATGCTGAGGACAGCCTTAAAAGCCCATGGATTTTATTTTTGAAATAGTC
GAATTTGAATGACATGTAGAGACTGAGCCAAAGTTAGTCACTTTTCCAAAAAATTCTCGAACAGGTCGTTTTTA
TTTTTTCGAATGTTACATTTTGACGTCATAGTGATATTTATGATGTAAGAAAGATCTTTGTGTAATACTCTCAAT
GAGTTTTACAACTTTTTTATTTTGATTTCTTTGTTTTACAGTTTTTCTTGCTAGAATTTTTTGAATTTTTTATACTCA
TTTTATACTTATTCAAAACTATAAAAACGCCATCGTCGCATTGTCTCTCAAAATTAGCTGTTATAAATTATTCATG
CACTTCAAAAAATAATAGGTATAGTTATTTATTTAACAAGTTTGAGTGTAAATCGGACGTTTTATGTCACGAGT
GGATTATCAAACCACTATTTTACAATTTTTTATCAGTCTTATTTAAAAAAATAATTCGGTAAAATGCGAACTTGG
CGTTTTGAACAACTAATAGAACTCCATTTAAAATTAGTGATCCAGACTTCGTTCCTATATAACATAATTTCAAAA
TTTTTGACATTGATAATTGATTTAAAATGTAAAACAATAATCAAAAACCTGAGTTGTCATTAAAAAATTTAATAT
GATGTTATTACTCAAAAACGAATGACGTCAGGTCAAGTCAGGTCAGGTGATGACTTTATAGTGTCAAATTATAT
ATTTAAAAAAATAAAATCGACCTGTGCAAAAATTTTGACTCATTAGGAAGATACGATTTTGTGCGTTACTTTTA
GTTCAGTCTGTATTTTTTTAATTAATTAGGTCGAGTATTTTTTATCCATTCTCACTGTGAAAAATGTATTGGTAAA
AAAAATTTATTTAGAAATTTTTATCTTATGTTCGATAAATTTTTATTAGTTTATTTCCCCAAGCAATAAAAAAATT
GCTTCTACGATTCCTGCTTTTCTGCTTTTACTACATGATATCCAAGCATGCATTTACATTACGCTAAACTGACTCT
TTGCATTTTTTTTTATTTTAACGGTCGGTTTTAATGAGTCGGTTAGTAATTTAAAGTTTATTTAATTTTATGGATT
AGGGCCAATTTTCCGAGAAAAAGCCCATTTCCGCCGACTTCCGAATTTGTGTGAAAATCTTGCGATAGGTTCCG
TTTCCTGTATCGAATCCCCGTTCGTCCCCGTCGTTGTTTACCGTCCGATTCCCCTATGGGCAACAGCTGTGATCT
AAAAACCCACCCCTGGAACAGGGAGGGGTCGATTTATGTTACTGGTCTCGTGCCATGTGCGAAACAACGTTGA
GAATTTTTAGTTGCGTTCGATCTTTAGAAATACGCGCACGCGACACAATTCAACCCATCACCAATTACCCAACAT
CCGGTCCGGCATTGAAGTCATCCACTTAACAGATCTGAACTCTGACACAGACAGTAGTGAC

## *Tc-EF1-α-B* regulatory region

The following upstream regulatory sequence of the *Tc-EF1-α-B* gene drives neural expression in the pattern shown in red in Fig. 4c-e and supplementary figure S2 p-x.

CDS of upstream gene (NCBI)
5´start of ORF

Primers used for amplification:

| name | Tm | length | restriction | sequence (5´-3´) |
|---|---|---|---|---|
| TcEFb-F3 | 61C | 2246 | EcoRI | ggaattcCCATCACCGTCGAAACCATA |
| TcEFb-R2 | 59C | | BamHI | cgggatccGATTACGAGTTTTGCCTGGAA |

>ref|NW_001094328.1|TcaLGUn_WGA243_1:19....-194948 (gene LOC658093 = hmm350)
GACCCAGAAACTTAATCTGGTGGCTCGGGGCGACCCGGGCATTCGGGGGTGGACCAACTACCTTCCTTTG
TGGCCATCACCGTCGAAACCATAATTATTAATTGTTATGTAAATATTGCTAAATAAATTATTTCTTATTC
GCGTTTTAATTGCGTTTTTGAATTGAATCTCCCGCGCCGATTGCAACCCCGGCGACGTCACATCCCTTGA
TTTTTTTCGCGATCTCATTGGTTCAGTAATTTTAGACACTTCCCCTATTTCCCCAAAATTTGCATTTCCT
GTGAAAATTAATTGCGACTTTTGGCCCAAACTGCGTAAATCCCACTATTTTAGCACCGTTATTGGGCCAA
AAATGCAAAACGGTTTAAAAATGCCCTATTTCGCGTTGATCGTAAATCAATATCCTATCCCCACCATCCA
GATTCTCCTTCTATTTCCGTTTGCTCGCGGCTAGGAGAGGATTGTCGTTTTTCCCGTTCTCTTGCTGCAG
TTTCAGAGGTAAGTGGAGCTTTCCCGATTTTTTACAAATTTTATCAGTTATCAGTCTTTAAAATATTCAT
CCGGTGTGTGTGACAATCGCATAAATTCCCTCAAATCACGGAAAACTGCACTAGGGAGGTATTTTCTTTG
ATTATTCGAAATTTTGGCAGATTTTCTCGCCGGCACTTTGCATCGCCTACTTTTCGACTTTTCGCCCAAA
AGGCACTTTTTGCAATGTGTCAAATTCTGACAATCGATGATTTGTAAAAAAAAACAATATTTTTTGCAAA
AAATAAACAAAATTCCTCGTGTCAGTTGAAAATTGTGCGAGCCGGTATGTATTTTCCATTGAATTATTTT
GTTGCATCAGATTTGCAAATGAATGAGTCAGTTTAAATCCCAGAATTGCGGTTTTTCGACCTACCTCAAT
AACGATTTTGCTCCTCGAGTCAATAACCAAAACCGGATTGCCAGTGGGGGAGTTAATCGATCGGCCAAAT
TTATTTGGGATTTTTTGATAATATTGATTTAAATGGATTGCAACACAAAATGGCCGCCCTATTGTTCAAA
GCTGCATTTCTTAGCCGGCCGGCGGCCCGGAGGCACTTAATTTTTTAACAATAAGTCAATTTCGGAATGG
CAATAACACGGGTTCAACGACCTCACAGTTTCACTATTAGATTTTTACCTGTTTGATAAAAAACCAACTG
CATTGTTTTGCAGCGGTTTATTTTCCTGTTTGGCTAATTGAGTCAAGGTCGTGGAACACCGTTAGTTCCA
GTGCTTGTAAACACATGATTTTCGAGTCAGGGACTCGGCATTAGGCCCCAGATACGATTTCAAATCTATC
TAGTATTTGGCTGGCCTTATTGATCACCTGAACAGAACCTGTGAAGGTTTTACCTAATCTAAGGTTCAAG
GGCTGTATAGCCAGAACATTCATCCGTTTTAAAATTGTAAAAATACCTTTCTATATCACCCAATTTTAAT
ATCTTTACTAATAATAAATAATTCAAATTCTAGGTACTAAAGAATCATAATTCCGCATGATTCACTCACT
GCATACCTAAGTTTTTATTCACTTTGATATATCAAAATTAATCGCTTTTAATTCTGGTTTAAGTTCATAG
CCAGTGGCGTATTTAGGTTCATTGCTTCTCAGGGATACTTAATTTTGCCGTCCCTGCATATATTTCTTTA
AAAAATTGTCGTGCTTTGGAAATTTGTAGCACCATTCGTATTTTCAAAATTTTAAATGGTACCACCCTTC
TTGTGCCAAATGAACTACTCGAAAAATGCAAAATTAACTTTAAATACCTGAAAAAATTGAAAAAAAACTG
AAAAAATGCACTAGATTTTTAAATCTTCCCTTTATTTCTATTAAAAAATACCCTGTATTTATTTAAGAAA
GTTTTTGTTTCTGAAAAACCATAGCCATTTTTACACTTTTTTTATGTTATCGCTGACTTTTTTCAAAGAT
GTGTACTGTTCAGGCTTTCTCTCATCACTCGAGGTTTAAGTCCACTCGTGGAATAAAGAGTCCGATGTTA
TTCACTCAGGCTCGTTAAATAAATAACTATATTAACAAACTAGCAATATTCGACAGTAAATTAAATTCAA
TAACGTGGCAAACAAAAGAGCAACAATTAGGGCGGTTCCTACCGAAAAAATTAAAAAAGTTGCTTGGAAA
GAAATTTTGAATCATTTAAAAAACACTGCGTTATTTGAGTTGTAATGCAAAAGTTTTTAGAGCCAATTTT
TGGTGGGGGTTCACACCACCCTCACAGAGTGTGTGGGCGCCGCTGACGTTTGCTCTTTTTCCAGGCAAAA
CTCGTAATCCGCCATGGGTAAGGAAAAGATCCATATTAACATCGTCGTG

# Material and Methods:

## Beetle strains:

The “mushroom body green” line is an enhancer trap found in the GEKU screen [7]. The “glia blue” line was generated by driving ECFP with 6 copies of the artificial P3 Promoter using the *Tc-hsp68* core promoter. The “neuron red” line was generated by Michalis Averof by driving dsRedExpress using the regulatory sequence of the *elongation factor 1 alpha-B (Tc-EFB)* gene, which has two copies in *Tribolium*. While Tc-EFA drives ubiquitously as expected [8] the *Tc-EFB* enhancer unexpectedly drove in neural cells. The “brainy” line was generated by crossing these two lines. By sibling crossings, these lines were made homozygous.

## Immunohistochemistry:

The fluorescence signal of the brainy line enabled us to dissect the brain of the first instar larvae (L1) by using a fluorescence binocular (SteREO Lumar. V12; Zeiss, Jena, Germany) equipped with a NeoLumar S 1.5x FWD 30 mm ocular (Zeiss). Brains were dissected in a concavity slide in cold PBS (phosphate-buffered saline, 0.01 M, pH 7.4) and fixed in 4% formaldehyde (FA, Roth, Karlsruhe, Germany) in PBS (for up to 60 min on ice or up to 3 h at room temperature (RT)). Brains were rinsed in PBS 3 x 10 min at RT. Transfer into different solutions was performed by a pipette (0.1 – 2.5 µl; Eppendorf, Hamburg, Germany). Preincubation was done for about 1 h on ice in 5% normal goat serum (NGS; Jackson ImmunoResearch, Westgrove, PA, USA) in PBS containing 0,3% Triton X-100 (PBST; Sigma-Aldrich, Steinheim, Germany). We incubated the brains over night at 4°C in a 1:50 dilution of the synapsin antibody in PBST containing 2% NGS in combination with one of the rabbit antisera (anti 5HT, 1:10000; anti MIP: 1:5000, anti AT: 1:5000, anti TKRP, 1:50000 in L1 or 1:20000 in adult; anti PVK, 1:4000; anti DC0: 1:200; or Repo: 1:500). For the anatomical studies we also used Alexa Fluor 488-coupled phalloidin (phalloidin; Molecular Probes, Eugene, OR, USA). The brains were rinsed 3 x 10 min with PBST, afterwards they were incubated with the secondary goat anti-mouse conjugated to Cy5 (GAM Cy5) and goat anti-rabbit antibody conjugated to Cy3 (GAR Cy5), or GAM Cy3 and GAR Cy5 (each 1:300, Jackson ImmunoResearch, Westgrove, PA, USA) in PBST and 1% NGS for 4 – 6 h at RT or over night at 4°C. Subsequently the brains were rinsed again with PBST 3 x 10 min and were finally mounted in 80 % glycerol (Sigma-Aldrich) and 20 % PBS. To prevent the brains from compression, we mounted them between two coverslips using one reinforcing ring as spacer (Zweckform, Oberlaindern, Germany).

Adult brains were treated according to the protocol published in Binzer et al. (2013). In short, adult brains were dissected in cold PBS, fixed in 4% FA in PBS over night at 4°C. Subsequently washed 5 x 10 min with PBS at RT and were preincubated with PBST containing 5 % NGS for 1-2 days. Afterwards brains were incubated with primary antibody (see above) for 2-3 days, washed 5 x 10 min with PBST at RT and incubated in secondary antibody (see above) for 2 days at 4°C. Subsequently we washed the brains 5 x 10 min with PBS at RT followed by dehydrated in an ascending ethanol series (50%, 70%, 90%, 95%, and two times 100%, for 2.5 min each) and then cleared in methyl salicylate (Merck, Gernsheim, Germany), until the tissue was transparent. Finally brains were mounted in Permount (Fisher Scientific, Pittsburgh, PA, USA) using two reinforcing rings as spacers between two coverslips.

Primary and secondary Antibodies and tissue markers:

All used antisera are highly conserved among insects (neuropeptides: [9–11]; serotonin: [12]; DC0: [13]; Repo: [14]. The antibodies against neuropeptides and synapsin were tested in *Tribolium castaneum* by [1,15]. The monoclonal primary antibody from mouse against a fusion protein consisting

of a glutathione-S-transferase and the first amino acids of the presynaptic vesicle protein synapsin I coded by its 5′-end (SYNORF1; 3C11, #151101), was used to selectively label neuropilar areas, especially for 3D reconstructions of the brain. It was kindly provided by Dr. Erich Buchner (University of Würzburg, Germany) and was first described by Klagges et al. (1996). The antibody was used at a dilution of 1:50. The polyclonal antiserum against the biogenic amine serotonin (5HT) was raised in rabbit (DiaSorin, Kansas City, MO, USA).). It was used at a dilution of 1:10000. The antiserum against the neuropeptide *Manduca sexta* allatotropin (Mas-AT, pGFKNVEMMTARGFamide) was raised in rabbit. It was kindly provided by Dr. J. Veenstra (University of Bordeaux, Talence, France) and first described by [16]. The antibody was used at a dilution of 1:5000. The antiserum against the neuropeptide *Periplaneta americana* myoinhibitory 1 (Pea-MIP1, GWQDLQGGWamide) was raised in rabbit. The antiserum was kindly provided by Dr. H. Agricola (University of Jena, Germany) and has been described by [17]. It was used at a dilution of 1:5000.

The antiserum against the neuropeptide *Locusta migratoria* Tachykinin 2 (Lom-TK2, APLSGFYGVRamide) was raised in rabbit. It was kindly provided by Dr. H. Agricola (University of Jena, Germany) and first described by [18]. The antibody was used at a dilution of 1:20000 in adults or 50000 in larva.

The antiserum against the neuropeptide *Periplaneta-americana* Periviscerokinin 2 (Pea-PVK2, GSSSGLISMPRVamide) was raised in rabbit, The Antiserum was kindly donated by Dr. M. Eckert (University of Jena, Germany) and first described by [19].

The DC0 antiserum recognizes the catalytic subunit of the protein kinase A (PKAc) of the vinegar fly *D. melanogaster*. Anti-DC0 was raised in rabbit and was kindly provided by Dr. D. Kalderon (Columbia University, NY, USA). It was first described by [20]. We used this antiserum in a concentration of 1:200).

The Reversed-Polarity (4α3) (Repo) antiserum is directed against the transcription factor Reversed-Polarity of *D. melanogaster* and was used to visualize somata of glia cells (Halter et al., 1995). It was raised in rabbit and kindly provided by Dr. B. Altenhein (University of Mainz, Germany) and had been described by Halter et al. (1995). It was used in a dilution of 1:500.

As secondary antibody we used goat anti-mouse conjugated to Cy5 (GAM Cy5) and goat anti-rabbit antibody conjugated to Cy3 (GAR Cy5), or GAM Cy3 and GAR Cy5 (all Jackson ImmunoResearch, Westgrove, PA, USA). Each secondary antiserum was used in a dilution of 1:300. Alexa Fluor 488-coupled phalloidin (phalloidin; Molecular Probes, Eugene, OR, USA) was used to visualize F-actin of axons, to label the whole brain structure. It was used at a dilution of 1:200.

**Data analysis and three-dimensional reconstruction:**

Fluorescence signals were scanned with a confocal laser scanning microscope (Leica TCS SP5 CLSM; Leica, Bensheim, Germany) equipped with a 63× glycerol objective (HCX PL APO 63x/1.30 Glyc 21°C CS (working distance: 0.26 mm); Leica, Bensheim, Germany) or 40x oil objective (HCX PL APO 40x/1.25-0.75 Oil Lbd. bl. (working distance: 0.1 mm); Leica) at a resolution of 1024x1024 or 512x512 pixel, a pinhole of 1 Airy unit, a scanning speed of 400 or 200 Hz, a step size of 0.5–1.0 µm, a line average of 2–4, and a digital zoom of 1.0–2.5.

Images taken from AMIRA 5.1 or 5.2 (FEI, Hillsboro, OR, USA) were arranged and cropped by using CorelDRAW X3 (Corel, Ottawa, Ontario, CA). 3D reconstructions of brains were performed by using the

segmentation editor and the polygonal surface model in AMIRA, based on stainings with the synapsin antibody or alexa Fluor 488-coupled phalloidin, according to Kurylas et al. 2008 [7].

**Signal colocalization and cell counting:**

Larval brains were scanned with a step size of 0.5 µm (see above) resulting in a stack of appr. 80 – 90 optical sections. Using the model “ProjectionView” in AMIRA 5.1 or 5.2, we merged 10 successive sections to one maximum intensity projection, respectively, in which we counted the cell numbers and colocalization of the signals.

**<u>Orientation of the adult and larval brain</u>**

To compare adult and larval brains, we used the neuraxis instead of the body axis for orientation [21].

**References:**


1. Binzer, M., Heuer, C. M., Kollmann, M., Kahnt, J., Hauser, F., Grimmelikhuijzen, C. J. P. & Schachtner, J. 2014 Neuropeptidome of Tribolium castaneum antennal lobes and mushroom bodies. *J. Comp. Neurol.* **522**, 337–357. (doi:10.1002/cne.23399)
2. Heuer, C. M., Kollmann, M., Binzer, M. & Schachtner, J. 2012 Neuropeptides in insect mushroom bodies. *Arthropod Struct. Dev.* **41**, 199–226. (doi:10.1016/j.asd.2012.02.005)
3. Wegerhoff, R., Breidbach, O. & Lobemeier, M. 1996 Development of locustatachykinin immunopositive neurons in the central complex of the beetle Tenebrio molitor. *J. Comp. Neurol.* **375**, 157–166. (doi:10.1002/(SICI)1096-9861(19961104)375:1<157::AID-CNE10>3.0.CO;2-S)
4. Wegerhoff, R. & Breidbach, O. 1992 Structure and development of the larval central complex in a holometabolous insect, the beetle Tenebrio molitor. *Cell Tissue Res* **268**, 341–358.
5. Zhao, X., Coptis, V. & Farris, S. M. 2008 Metamorphosis and adult development of the mushroom bodies of the red flour beetle, Tribolium castaneum. *Dev. Neurobiol.* **68**, 1487–1502. (doi:10.1002/dneu.20669)
6. Lin, H.-H., Lai, J. S.-Y., Chin, A.-L., Chen, Y.-C. & Chiang, A.-S. 2007 A Map of Olfactory Representation in the Drosophila Mushroom Body. *Cell* **128**, 1205–1217. (doi:10.1016/j.cell.2007.03.006)
7. Trauner, J., Schinko, J., Lorenzen, M. D., Shippy, T. D., Wimmer, E. A., Beeman, R. W., Klingler, M., Bucher, G. & Brown, S. J. 2009 Large-scale insertional mutagenesis of a coleopteran stored grain pest, the red flour beetle Tribolium castaneum, identifies embryonic lethal mutations and enhancer traps. *BMC Biol* **7**, 73.
8. Sarrazin, A. F., Peel, A. D. & Averof, M. 2012 A Segmentation Clock with Two-Segment Periodicity in Insects. *Science*
9. Nässel, D. R. 2002 Neuropeptides in the nervous system of Drosophila and other insects: multiple roles as neuromodulators and neurohormones. *Prog. Neurobiol.* **68**, 1–84.
10. Homberg, U. 2002 Neurotransmitters and neuropeptides in the brain of the locust. *Microsc. Res. Tech.* **56**, 189–209. (doi:10.1002/jemt.10024)
11. Nässel, D. R. & Homberg, U. 2006 Neuropeptides in interneurons of the insect brain. *Cell Tissue Res.* **326**, 1–24. (doi:10.1007/s00441-006-0210-8)
12. Dacks, A. M., Christensen, T. A. & Hildebrand, J. G. 2006 Phylogeny of a serotonin-immunoreactive neuron in the primary olfactory center of the insect brain. *J. Comp. Neurol.* **498**, 727–746. (doi:10.1002/cne.21076)
13. Farris, S. M. & Strausfeld, N. J. 2003 A unique mushroom body substructure common to basal cockroaches and to termites. *J. Comp. Neurol.* **456**, 305–320. (doi:10.1002/cne.10517)
14. Halter, D. A., Urban, J., Rickert, C., Ner, S. S., Ito, K., Travers, A. A. & Technau, G. M. 1995 The homeobox gene repo is required for the differentiation and maintenance of glia function in the embryonic nervous system of Drosophila melanogaster. *Dev. Camb. Engl.* **121**, 317–332.
15. Utz, S., Huetteroth, W., Vömel, M. & Schachtner, J. 2008 Mas-allatotropin in the developing antennal lobe of the sphinx moth Manduca sexta: Distribution, time course, developmental regulation, and colocalization with other neuropeptides. *Dev. Neurobiol.* **68**, 123–142. (doi:10.1002/dneu.20579)
16. Veenstra, J. A. & Hagedorn, H. H. 1993 Sensitive enzyme immunoassay for Manduca allatotropin and the existence of an allatotropin-immunoreactive peptide in Periplaneta americana. *Arch. Insect Biochem. Physiol.* **23**, 99–109. (doi:10.1002/arch.940230302)
17. Predel, R., Rapus, J. & Eckert, M. 2001 Myoinhibitory neuropeptides in the American cockroach. *Peptides* **22**, 199–208.
18. Veenstra, J. A., Lau, G. W., Agricola, H. J. & Petzel, D. H. 1995 Immunohistological localization of regulatory peptides in the midgut of the female mosquito Aedes aegypti. *Histochem. Cell Biol.* **104**, 337–347.

19. Eckert, M., Herbert, Z., Pollák, E., Molnár, L. & Predel, R. 2002 Identical cellular distribution of all abundant neuropeptides in the major abdominal neurohemal system of an insect (Periplaneta americana). *J. Comp. Neurol.* **452**, 264–275. (doi:10.1002/cne.10382)
20. Lane, M. E. & Kalderon, D. 1993 Genetic investigation of cAMP-dependent protein kinase function in Drosophila development. *Genes Dev.* **7**, 1229–1243.
21. Ito, K. et al. 2014 A systematic nomenclature for the insect brain. *Neuron* **81**, 755–765. (doi:10.1016/j.neuron.2013.12.017)
