# Supplementary material for: The insect central complex as model for heterochronic brain development—background, concepts, and tools
Source: Dev Genes Evol. 2016 Apr 7;226:209–19. doi: 10.1007/s00427-016-0542-7 (PMC4896989; doi:10.1007/s00427-016-0542-7)
Supplement: Supplementary file 2 — Analysis of Tc-six3 phenotype by immunohistochemistry (PDF 1117 kb) [file 427_2016_542_MOESM2_ESM.pdf]

Supplementary Figures:

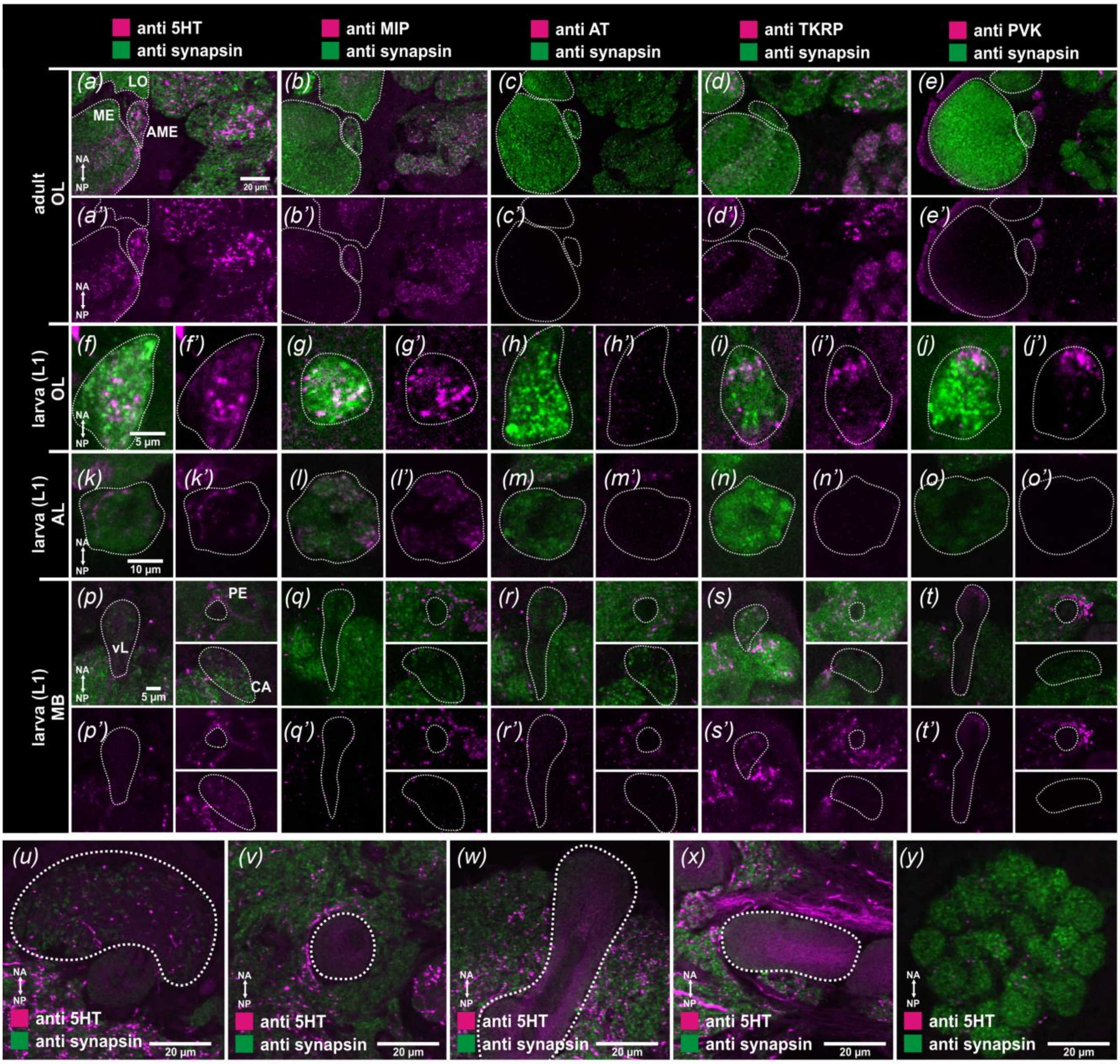

Supplementary figure S1 Comparison of brain areas between adults and first instar larvae of *Tribolium* Immunostainings of adult and first instar larval (L1) brains of *Tribolium* of three of the four main neuropils: (a-j and a'-j') optic lobes (OL); (k-o and k'-o') antennal lobes (AL) and (p-t and p'-t') mushroom body (MB). An antibody against synapsin (green) was used in combination with one additional antibody against a neuromediator (5HT: serotonin, MIP: Myoinhibitory Peptide , AT: Allatotropin, TKRP: Tachykinin related peptide, PVK: Periviscerokinin) (magenta). AME: accessory medulla, CA: calyx, LO: lobula, ME: medulla, mL: median lobe, PE: pedunculus, and vL: ventral lobe. Scale bars, orientation bars (NA: neuraxis anterior and NP: neuraxis posterior), labeling of the neuropils and scale bars in the left column of the line applies for all pictures of the line. *(u-y)* Immunostainings of neuropils from the adult animal against 5HT and synapsin of the Ca *(u)*, the PE *(v)*, the vL *(w)*, the median lobe *(x)*, and the AL *(y).*

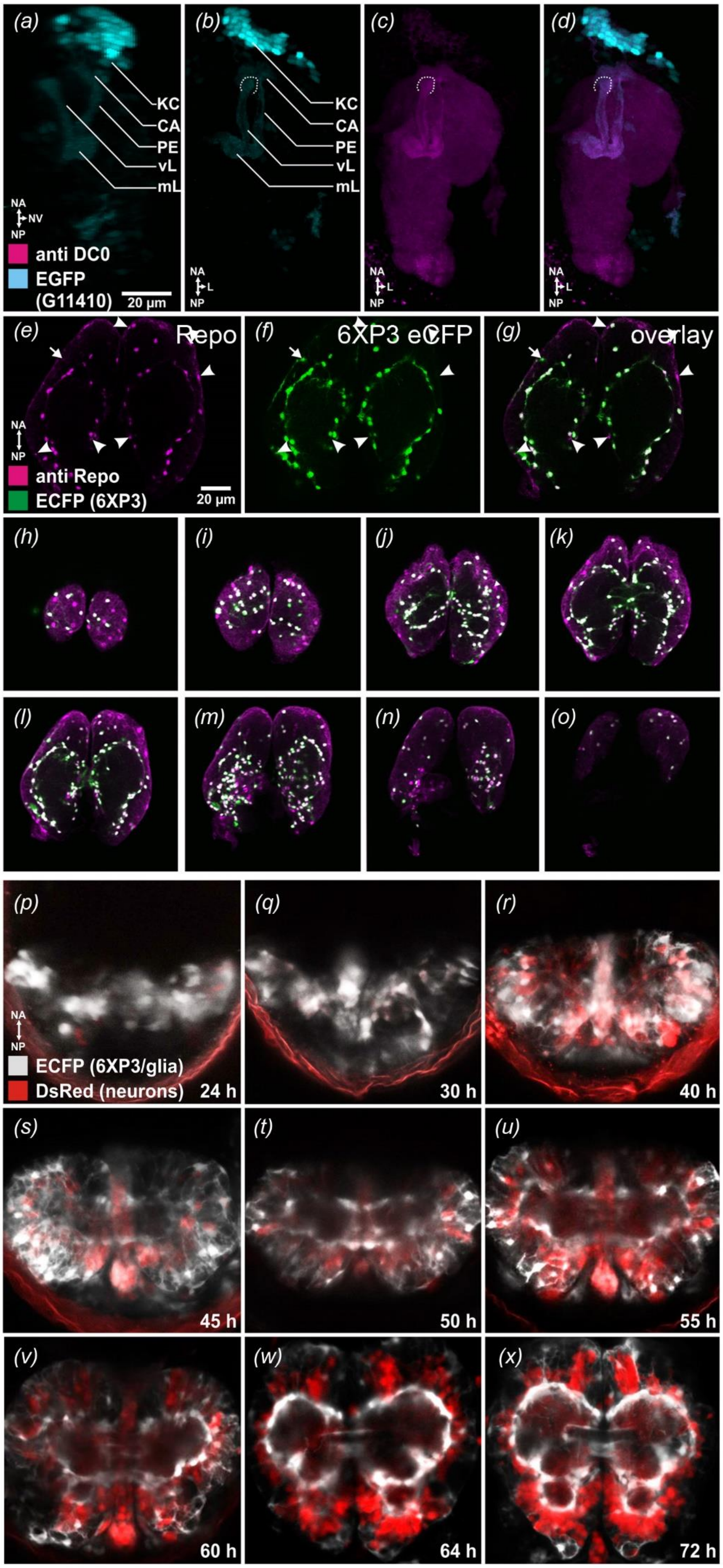

Supplementary figure S2

Characterization of the MB-green and the brainy line

(a-d) Characterization of the enhancer trap line G11410 (MB-green), which marks the MB. *(a,b)* One MB shown from two different angles to display all details. Labelling as in figure 3, KC: Kenyon cells. *(c,d)* Colocalization of the EGFP signal of G11410 with the DC0 antibody confirms labelling of the MB. Note that the KC are marked in G11410 but not by the DC0 antibody. *(e-o)* Characterization of the glia-blue line (6XP3), which marks glia with ECFP (green) in combination with an antibody against *Drosophila* Repo (magenta). Overlay of both signals *(g-o)*. Most cell bodies marked with ECFP are Repo positive confirming glia-specific staining. Note that some glia cells are not marked by glia-blue (white arrowheads in *e-g*) and some ECFP marked cells are not Tc-Repo positive (arrows in e-g). *(p-x)* Embryonic developmental series of fluorescence signal in the brainy-line. Orientation bars in *(e)* and *(p)* apply for *f-o* and *q-x*, respectively).

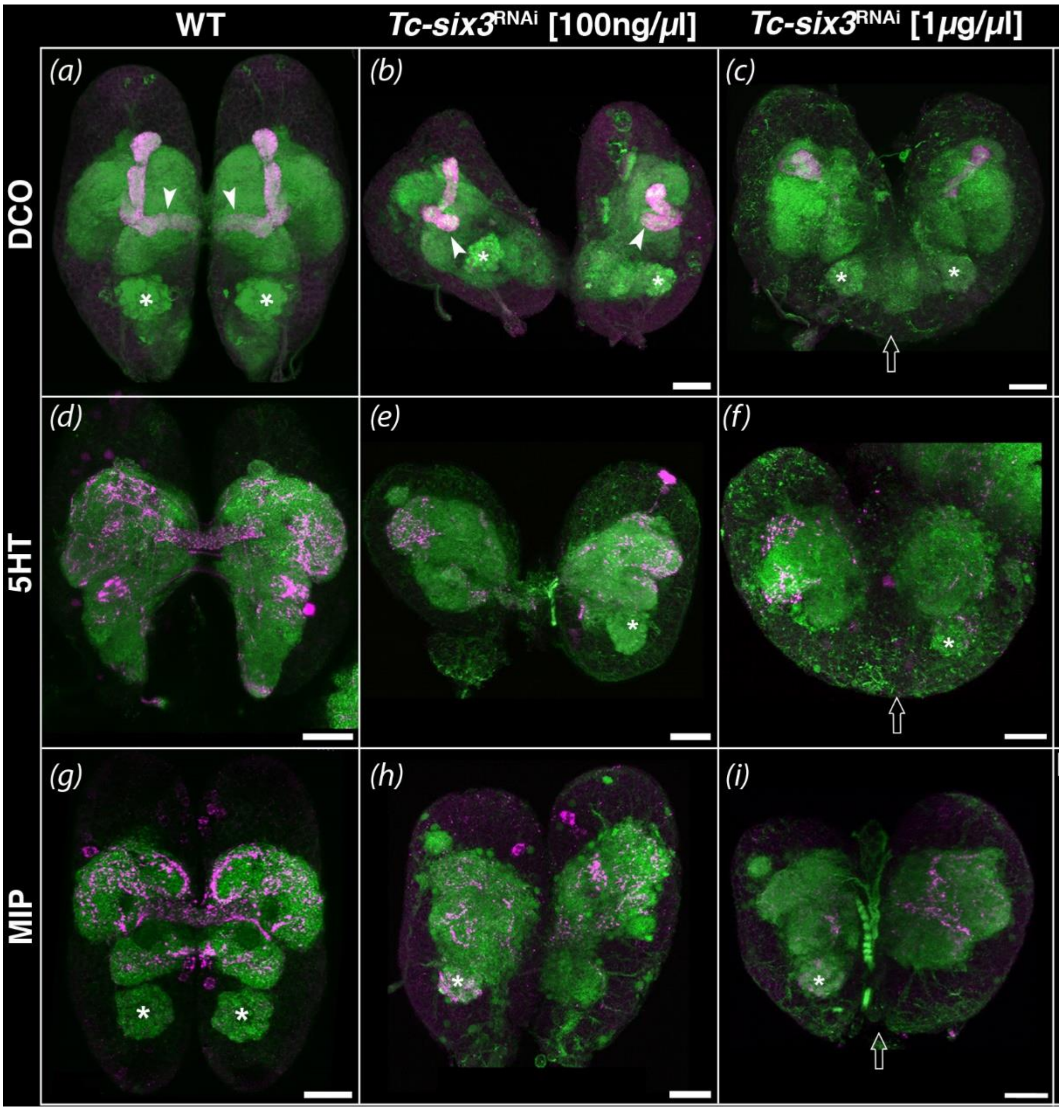

Supplementary Figure S3 Analysis of the *Tc-six3* phenotype by immunohistochemistry

(a,d,g) L1 wildtype brains stained for DC0 *(a)*, serotonin (5HT) *(d)*, and myoinhibitory protein (MIP) *(g)*. *(b,c,e,f,h,i)* Brains of late *Tc-six3* RNAi knock-down embryos. Anterior is up and phalloidin staining is shown in green in all panels. In mild RNAi treatments (100 ng/µl), the mushroom bodies are still present but the median lobes do not touch at the midline (compare location of arrowheads in *(a,b)*). The CBU is marked by serotonin and MIP, but is absent in RNAi embryos *(e,h)*. With higher RNAi concentrations (1 µg/µl), the phenotype gets stronger and the midline of the brain becomes more severely affected (open arrow in *(c,f,i)*). Scale bars depict 20 µm.
